# Risk factors for lymph node metastasis and indication of local resection in duodenal neuroendocrine tumors

**DOI:** 10.1002/jgh3.12718

**Published:** 2022-02-17

**Authors:** Eisuke Nakao, Ken Namikawa, Toshiaki Hirasawa, Kaoru Nakano, Yoshitaka Tokai, Shoichi Yoshimizu, Yusuke Horiuchi, Akiyoshi Ishiyama, Toshiyuki Yoshio, Souya Nunobe, Junko Fujisaki

**Affiliations:** ^1^ Department of Gastroenterology Cancer Institute Hospital, Japanese Foundation for Cancer Research Tokyo Japan; ^2^ Department of Pathology Cancer Institute Hospital, Japanese Foundation for Cancer Research Tokyo Japan; ^3^ Department of Gastroenterological Surgery Cancer Institute Hospital, Japanese Foundation for Cancer Research Tokyo Japan

**Keywords:** duodenal neuroendocrine tumors, endoscopic resection, laparoscopy and endoscopy cooperative surgery, lymph node metastasis

## Abstract

**Background and Aim:**

The risk factors for lymph node metastasis (LNM) of duodenal neuroendocrine tumors (DNETs) are not well identified, and a definitive standard of treatment for DNETs has not been established. In this study, we aimed to identify the risk factors for LNM and establish the indication of local resection for DNETs.

**Methods:**

We retrospectively reviewed 55 patients with 60 non‐ampullary and nonfunctional DNETs. We evaluated the risk factors for LNM and compared the outcomes between endoscopic resection (ER) for DNETs <5 mm and laparoscopy and endoscopy cooperative surgery (LECS) for DNETs ≥5 mm.

**Results:**

LNM was present in four (8.7%) patients. Univariate analysis revealed that tumor size ≥10 mm, positive lymphovascular invasion (LVI), and 0‐Is morphology were significantly associated with LNM (*P* = 0.008, *P* = 0.037, and *P* = 0.045, respectively). ER and LECS were performed for 18 and 11 DNETs, respectively. All lesions treated with ER or LECS were confined to the submucosal layer. The median tumor size was 3 mm in ER and 6 mm in LECS. Although there was no significant difference in the R0 (no residual tumor) resection rate, R0 resection was completely achieved in the LECS. No significant differences were observed in terms of complication rates. No recurrence was observed in any of the groups.

**Conclusions:**

Tumor size ≥10 mm, positive LVI, and 0‐Is morphology were significant risk factors for LNM. We demonstrated that ER is feasible and could be safely applied for DNETs <5 mm, and LECS could be applied for DNETs 5–10 mm in size.

## Graphical abstract

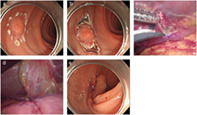

## Introduction

Duodenal neuroendocrine tumors (DNETs) are observed less frequently than rectal and gastric neuroendocrine tumors and account for only 16.7% of all gastrointestinal neuroendocrine tumors in Japan.^1^ Their prevalence is much lower in the United States, accounting for only 2–3%.^2^, ^3^ While the likelihood of detecting small DNETs is higher than for other tumors due to the development of high‐resolution imaging and screening gastrointestinal endoscopy,^4^ these tumors remain rare.

The therapeutic approach for DNETs is determined by the tumor size, location, histopathological grade, and stage. In addition, the risk factors for lymph node metastasis (LNM) should be considered in the selection of treatment methods. However, a definitive treatment standard for DNETs has not yet been established. According to the European Neuroendocrine Tumor Society (ENETS) guidelines, endoscopic resection (ER) is recommended for patients with DNETs <10 mm in size and limited to the submucosal layer.^5^ However, the application of ER for DNETs is not clearly described in the National Comprehensive Cancer Network (NCCN) guidelines^6^ or the Japan Neuroendocrine Tumor Society (JNETS) guidelines^7^ due to the lack of evidence.

Recently, the efficacy of ER for duodenal tumors has been reported.^8^, ^9^ In addition to ER, laparoscopy and endoscopy cooperative surgery (LECS) has been applied for the treatment of duodenal tumors for the purposes of safety and complete *en bloc* resection.^10^, ^11^ These less invasive treatments are considered adequate for DNETs <10 mm in size and limited to the submucosal layer with a low frequency of LNM and distant metastasis. However, there are only a few published studies investigating the efficacy of these local resections for DNETs,^12^, ^13^, ^14^ and no definitive criteria are available to discern whether ER or LECS is indicated for the local resection of DNETs.

In this work, we aimed to investigate the clinicopathological features of DNETs with a relatively large number of cases and identify the risk factors for LNM. In addition, we wanted to compare the short‐ and long‐term outcomes of ER and LECS to establish the application of local resection for DNETs.

## Methods

### Study design

Fifty‐five patients with 60 DNETs diagnosed at the Japanese Foundation for Cancer Research between January 2000 and December 2020 were enrolled. Ampullary and functional DNETs were excluded from the study. All lesions were histopathologically diagnosed by endoscopic biopsies or resection based on the findings from hematoxylin and eosin (HE) staining and immunohistochemical staining for chromogranin A and synaptophysin. The treatment methods for DNETs in this study were classified as surgery with lymph node dissection and local resection. The indications for local resection were as follows for lesions: <10 mm, limited to the submucosal layer based on endoscopic ultrasound (EUS), and without LNM or distant metastasis based on computed tomography (CT) findings. DNETs <5 mm were treated with ER and those of 5–10 mm were treated with LECS. If the lesion was completely removed by biopsy, it was defined as removal biopsy. Segmental resection was performed as a method of partial resection of the duodenum with regional lymph node dissection. Clinicopathological information was retrospectively collected from the hospital database, including the patients' backgrounds, tumor characteristics, and treatment outcomes. The short‐term outcomes of local resection were evaluated as follows: procedural time, R0 (the classification of no remaining tumor) resection rate, complication rate, time to first oral intake, and length of stay. The long‐term outcome of local resection was evaluated by the recurrence rate. Tumor size was assessed by histopathological findings in surgically or endoscopically resected cases and by endoscopic findings in cases that underwent biopsy. The morphology of DNETs was classified according to the Paris classification^15^ based on endoscopic findings. Lymphovascular invasion (LVI) was evaluated for all lesions based on the finding of HE staining, and of those invaded submucosal layer or deeper were additionally examined with D2‐40 immunohistochemistry and elastic fiber staining. The World Health Organization (WHO) 2019 classification of tumors^16^ was used for the classification of histopathological grading assessed by the Ki‐67 index. Clavien–Dindo Grade^17^ was applied to evaluate perioperative complications, and those higher than grade II were defined as clinically significant complications. Endoscopic examinations with or without CT were performed at 6 or 12 months after treatment, and every year thereafter, to check for local recurrence, LNM, and distant metastasis.

This study protocol was approved by the institutional review board of the Japanese Foundation for Cancer Research (IRB number: 2020‐1112).

### Endoscopic resection

Each ER was performed using one of the three methods: endoscopic mucosal resection (EMR), EMR with ligation device (EMR‐L), and EMR with a cap (EMR‐C). All these procedures were performed under intravenous conscious sedation (midazolam and/or petidine) with a single‐channel endoscope (GIF‐Q260J, Olympus, Japan) in the endoscopy room. Hyaluronichyaluronic acid (MucoUp, Boston Scientific, USA) or glycerin was injected to thicken the submucosal layer before each ER. For EMR, the lesion was resected using a snare. EMR‐L was performed by aspiration of the lesion into a ligation device, followed by placement of a ligation band. In addition to EMR‐L, EMR‐C was performed by aspirating the lesion into the attachment. After these procedures, the lesion was resected using a snare. An ESG‐100 (Olympus) or a VIO300D (ERBE, Germany) was used as the electrosurgical unit. The procedural time for ER was defined as the time from the start of submucosal injection to the completion of suturing mucosal defect.

### Laparoscopy and endoscopy cooperative surgery

**Figure 1:**
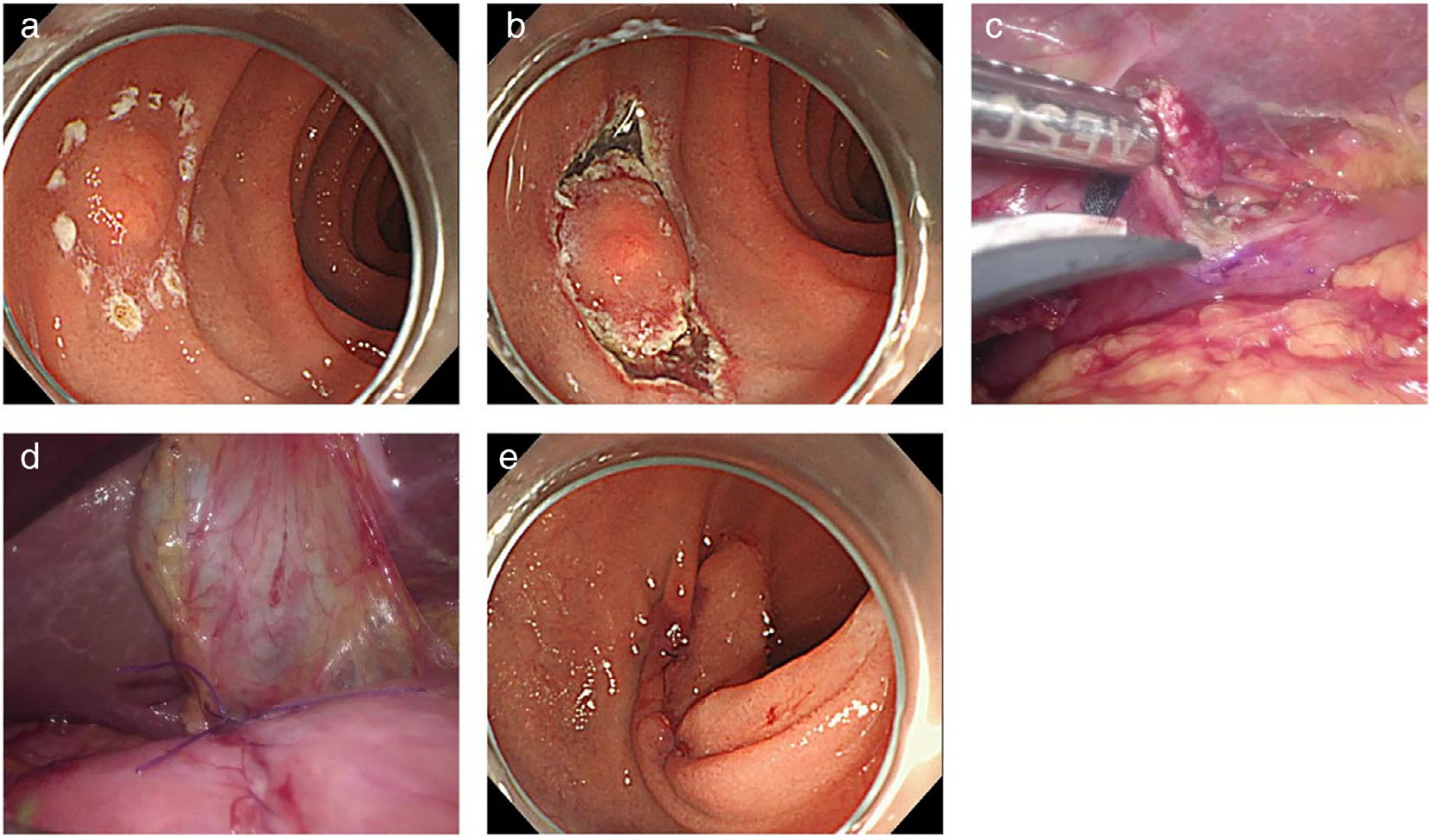
Laparoscopy and endoscopy cooperative surgery. (a) A duodenal neuroendocrine tumor with 6 mm diameter was observed at the superior duodenal angle. Circumferential marking was performed. (b) A circumferential incision was performed around the tumor with a dual knife. (c) The seromuscular layer was resected along the incision line using a laparoscopic approach to complete the full thickness resection. (d) The duodenal wall defect was closed using the laparoscopic suturing technique. (e) Intraluminal endoscopy confirmed that the duodenal wall defect was completely closed.

LECS was performed under general anesthesia in the operating room. Full thickness resection (FTR) was performed for complete tumor resection using the following procedure. First, endoscopists made a circumferential incision around the tumor with an IT Knife‐2 (Olympus) or a dual knife (Olympus) following submucosal injection using hyaluronic acid or glycerin. Then, a needle knife was used to perforate the seromuscular layer before the layer was resected along the incision line using a laparoscopic or endoscopic approach with the IT Knife‐2 to complete the FTR. EMR‐C was also performed as an FTR method. When performing EMR‐C, the lesion was fully aspirated to achieve FTR. Finally, the remaining duodenal wall defect was closed using the laparoscopic suturing technique with the assistance of intraluminal endoscopy. The procedural time for LECS was defined as the operation time. The details of the LECS are shown in Figure 1.

### Assessment of LNM

Forty‐six patients with DNETs who were followed up for at least 12 months were included in the assessment of LNM. In cases treated by surgical resection with lymph node dissection, LNM was assessed by histopathology and radiology during the follow‐up period. Other cases were assessed using radiology before and after treatment.

### Statistical analysis

All statistical analyses were performed with EZR,^18^ a modified version of the R commander software designed to add statistical functions that are frequently used in biostatistics. Statistical comparisons between both groups were conducted using the Fisher's exact test and the Mann–Whitney U test. The threshold for significance was set at *P* < 0.05.

## Results

### Clinicopathological characteristics of patients with DNETs

Table 1 shows the clinicopathological characteristics of the patients with DNETs. Thirty‐one of the 55 patients were men. The median age was 63 (range: 38–81) years. Among the patients, 23 underwent surgery and 27 underwent local resection; the remaining 5 had their lesion completely removed by biopsy. LNM was present in four patients, and no distant metastasis was observed. Liver metastasis recurrence occurred in one patient at the follow‐up period of 39.8 months. The details of the patient with liver metastasis recurrence are shown in Table 3.

**Table 1:** Clinicopathological characteristics of patients with DNETs

Patient characteristics (*n* = 55)	
Male:Female	31:24
Age, median (range), years	63 (38–81)
Treatment methods, *n* (%)	
Surgery	23 (42)
DG	12 (22)
Segmental resection	8 (15)
PD	3 (5)
ER	18 (33)
LECS	9 (16)
Removal biopsy	5 (9)
Lymph node metastasis, *n* (%)	4 (7)
Distant metastasis, *n* (%)	0 (0)
Recurrence, *n* (%)	1 (2)
Follow‐up period, median (range), months	39.8 (1.2–186)

Tumor characteristics (*n* = 60)	
Size, median (range), mm	6 (2–24)
Location, *n* (%)	
Bulb	54 (90)
Descending part	6 (10)
Morphology, *n* (%)	
0‐Is	27 (45)
0‐IIa	33 (55)
Invasion depth, *n* (%)	
Mucosa	8 (13)
Submucosa	43 (72)
Muscularis propria	6 (10)
Subserosa	3 (5)
WHO classification, *n* (%)	
G1	54 (90)
G2	6 (10)
Lymphovascular invasion, *n* (%)	11 (18)
Lymphatic invasion	8 (13)
Venous invasion	6 (10)

DG, distal gastrectomy; ER, endoscopic resection; LECS, laparoscopy and endoscopy cooperative surgery; PD, pancreatoduodenectomy.

The median tumor size was 6 mm (range: 2–24 mm). Of the 60 tumors, 54 were located in the duodenal bulb and 6 were located in the descending part. All tumors were protruding type (0‐Is) or superficial elevated type (0‐IIa). Tumors were confined to the mucosa in 8 patients and to the submucosa in 43; they were extended to the muscularis propria in 6 and to the subserosa in 3. Fifty‐four tumors were classified as WHO G1, and six tumors were classified as WHO G2. LVI was observed in 11 tumors; lymphatic invasion was present in 8, and venous invasion was present in 6.

### Risk factors for LNM

LNM was observed in 4 (8.7%) of the 46 patients who met the LNM assessment criteria. A comparison between the LNM‐positive and LNM‐negative groups is shown in Table 2. In univariate analysis, the factors that were significantly associated with LNM were tumor size ≥10 mm, positive LVI, and 0‐Is morphology (*P* = 0.008, *P* = 0.037, and *P* = 0.045, respectively). The summary of the patients with LNM is shown in Table 3. The tumor size of all patients with LNM was ≥10 mm, and LVI was present in three of them. All tumors with LNM showed a 0‐Is appearance. Of the four tumors with LNM, two were confined to the submucosa and the other two were extended to the muscularis propria and subserosa.

**Table 2:** Risk factors for lymph node metastasis

	Lymph node metastasis		
	Negative (*n* = 42)	Positive (*n* = 4)	*P*‐value
Age			1
<60 years	17	2	
≥60 years	25	2	
Sex			0.62
Male	23	3	
Female	19	1	
Location			0.053
Bulb	39	2	
Non‐bulb	3	2	
Morphology			0.045
0‐Is	18	4	
0‐IIa	24	0	
Size			0.008
<10 mm	31	0	
≥10 mm	11	4	
Invasion depth			0.10
Mucosa or submucosa	37	2	
Muscularis propria or deeper	5	2	
WHO classification			0.44
G1	37	3	
G2	5	1	
Lymphovascular invasion			0.037
Positive	8	3	
Negative	34	1	

ER, endoscopic resection; LECS, laparoscopy and endoscopy cooperative surgery.

**Table 3:** Summary of patients with lymph node metastasis and patients with liver metastasis recurrence

Patients with lymph node metastasis									
Case	Age, year	Sex	Location	Size, mm	Morphology	Invasion depth	WHO classification	Lymphovascular invasion	Treatment method
1	72	Female	Descending	10	0‐Is	SM	G1	Negative	Segmental resection
2	36	Male	Descending	15	0‐Is	SM	G1	Positive (ly+/v−)	PD
3	68	Male	Bulb	18	0‐Is	MP	G1	Positive (ly+/v−)	DG
4	57	Male	Bulb	24	0‐Is	SS	G2	Positive (ly−/v+)	DG

Patients with liver metastasis recurrence									
Case	Age, year	Sex	Location	Size, mm	Morphology	Invasion depth	WHO classification	Lympho vascular invasion	Treatment method
1	48	Male	Bulb	16	0‐Is	MP	G2	Positive (ly+/v+)	DG

DG, distal gastrectomy; ly, lymphatic invasion; MP, muscularis propria; PD, pancreatoduodenectomy; SM, submucosa; SS, subserosa; v, venous invasion.

### Clinicopathological characteristics of patients with DNETs treated by local resection

Table 4 shows the comparison of the clinicopathological characteristics of patients with DNETs treated by local resection. ER was performed for 18 patients with 18 DNETs between 2000 and 2020, and LECS was performed for 9 patients with 11 DNETs between 2005 and 2020. There were no differences between the two groups in terms of age and sex. The median tumor size was 3 mm in the ER group and 6 mm in the LECS group. No differences were identified between the two groups in terms of tumor location, tumor morphology, invasion depth, WHO classification, and LVI.

**Table 4:** Clinicopathological characteristics of patients with DNETs treated by local resection

Patient characteristics	ER (*n* = 18)	LECS (*n* = 9)	Total (*n* = 27)	*P*‐value
Gender, *n* (%)				
Male	10 (56)	5 (56)	15 (56)	1
Female	8 (44)	4 (44)	12 (44)	
Age, median (range), years	66 (38–81)	66 (57–76)	66 (38–81)	0.99

Tumor characteristics	ER (*n* = 18)	LECS (*n* = 11)	Total (*n* = 29)	*P*‐value
Size, median (range), mm	3 (2–6)	6 (4–7)	5 (2–7)	<0.001
Location, *n* (%)				
Bulb	18 (100)	11 (100)	29 (100)	1
Morphology, *n* (%)				
0‐Is	2 (11)	3 (27)	5 (17)	0.34
0‐IIa	16 (89)	8 (73)	24 (83)	
Invasion depth, *n* (%)				
Mucosa or submucosa	18 (100)	11 (100)	29 (100)	1
WHO classification, *n* (%)				
G1	15 (83)	11 (100)	26 (90)	0.51
G2	3 (17)	0 (0)	3 (10)	
Lymphovascular invasion, *n* (%)				
Positive	0	2 (18)	2 (7)	0.14
Negative	18 (100)	9 (82)	27 (93)	

ER, endoscopic resection; LECS, laparoscopy and endoscopy cooperative surgery.

### Short‐term and long‐term outcomes of local resection

The short‐ and long‐term outcomes of local resection of DNETs are shown in Table 5. The procedural time was significantly longer in the LECS group than in the ER group. Although there was no significant difference in the R0 resection rate, R0 resection was completely achieved in the LECS group. No significant differences were observed in terms of complication rates. Two patients in the ER group experienced complications. Two patients experienced intraoperative perforation, which was successfully managed using endoscopic clipping. An abdominal abscess was observed in one patient in the LECS group after treatment. The time to first oral intake and length of hospital stay were significantly shorter in the ER group. No recurrence was observed in any of the groups.

**Table 5:** Short‐ and long‐term outcomes of local resection

Outcome	ER (*n* = 18)	LECS (*n* = 9)	Total (*n* = 27)	*P*‐value
Procedural time, median (range), min	15 (5–111)	150 (122–227)	33 (5–227)	<0.001
R0 resection, *n* (%)	16 (89)	9 (100)	25 (93)	0.54
Complication, *n* (%)	2 (11)	1 (11)	3 (11)	1
Intraoperative perforation	2 (11)	0 (0)	2 (7)	0.54
Abdominal abscess	0 (0)	1 (11)	1 (4)	0.33
Time to first oral intake, median (range), POD	2 (1–5)	3 (1–4)	2 (1–5)	0.035
Length of stay, median (range), POD	6 (3–9)	8 (6–29)	6 (3–29)	0.001
Follow‐up period, median (range), month	21 (1–186)	60 (2–122)	30 (1–186)	0.30
Recurrence, *n* (%)	0 (0)	0 (0)	0 (0)	NA

ER, endoscopic resection; LECS, laparoscopy and endoscopy cooperative surgery; POD, postoperative.

## Discussion

In this study, we retrospectively reviewed 55 patients with 60 DNETs, which is a relatively large number of cases considering that DNETs are rare tumors, and showed the clinicopathological features of DNETs except for ampullary or functional DNETs. To establish the application of local resection for DNETs, we evaluated the risk factors for LNM. Subsequently, we evaluated the safety and feasibility of ER and LECS for DNETs to establish an appropriate treatment method for the local resection of DNETs.

Consistent with a previous study,^3^ DNETs were found to be more frequent in males and the mean age of the patients was 63 years. Most DNETs were located in the bulb (90%), and all DNETs presented the appearance of submucosal tumors with either a 0‐Is (45%) or 0‐IIa (55%) morphology. The majority of DNETs were limited to the mucosa or submucosa (85%) and classified as WHO G1 (90%). These findings are consistent with those of previous studies.^3^, ^19^ The LNM rate (7%) was also comparable with that in a previous study (11%),^20^ which excluded ampullary DNETs. However, the LNM rate in this study could be underestimated as a result of the exclusion of ampullary and functional DNETs, which have a higher rate of metastasis.^21^, ^22^ On the other hand, the positive LVI rate (18%) was slightly higher.^20^ One reason could be that LVI was comprehensively evaluated using immunohistochemistry with D2‐40 antibody and special staining for elastic fiber. These staining procedures were routinely used to evaluate LVI when the tumor extended to the submucosal layer, especially on the locally resected specimens. Another reason could be that the specimens resected by ER or LECS were examined more closely than those resected by surgery. LVI evaluation of surgical specimens was performed using 5‐mm slices, as compared to that of ER specimens using 2‐mm slices or LECS specimens using 2–3‐mm slices, and hence could have been underestimated.

For the selection of treatment methods for DNETs, the most important consideration is the possibility of metastasis. There are a few studies that have investigated the risk factors for LNM in DNETs.^20^, ^23^ Park *et al*. demonstrated that the risk factors for LNM in DNETs include non‐bulb location, tumor size >10 mm, invasion beyond the submucosa, WHO grade G2, and positive LVI.^20^ Hatta *et al*. reported that tumor size >10 mm, WHO classification G2, multiple lesions, and positive LVI were risk factors for metastasis in a multicenter, retrospective study.^23^ In the current study, tumor size ≥10 mm, positive LVI, and 0‐Is morphology were risk factors of LNM (*P* = 0.008, *P* = 0.037, and I = 0.045, respectively); non‐bulb location was close to significance (*P* = 0.053). The LNM rate of DNETs ≥10 mm, which was the most significant risk factor, was 27%, which was comparable to that found in a previous study (36%).^20^ In addition, no LNM was observed in DNETs <10 mm. Based on these findings, local resection without lymph node dissection can be applied for DNETs <10 mm after investigating invasion depth and metastases by CT and EUS. Although there could be some discrepancy in the presence of LVI among pathologists, positive LVI has been reported to be a risk factor for LNM both in previous studies^20^, ^23^ and in this study. Additional surgery with lymph node dissection should be considered when a positive LVI is revealed after local resection. Tumor morphology was thought to be related to LNM; most DNETs show a subepithelial tumor‐like appearance, which indicates a correlation between their morphology and the tumor volume underlying the deep range of the mucosa. However, our findings suggested that it could contribute to the decision making for the treatment only to some extent, and that the treatment method cannot be determined solely by morphology, since this is relatively subjective and can result in inter‐observer differences among endoscopists.

In the JNETS guideline, treatment by surgery for DNETs ˃10 mm is recommended, but the application of ER for DNETs is not clearly mentioned.^7^ In the NCCN guidelines, ER is recommended for localized DNETs if feasible, but no definite criteria for the application of ER are described.^6^ According to the ENETS guidelines, DNETs ˃20 mm or DNETs of any size with LNM should be treated by surgical resection, and DNETs <10 mm in non‐ampullary locations and without metastases or functional hormonal syndromes can be treated by endoscopic techniques.^5^ At present, there are some discrepancies in the application of ER between these guidelines. Since EMR for DNETs is considered to have a high rate of positive margins,^13^ endoscopic submucosal dissection (ESD) has been applied recently for the treatment of DNETs to overcome such problems.^12^ While ESD of DNETs can achieve complete *en bloc* resection,^14^ it is associated with a higher rate of perioperative complications than EMR, such as bleeding and perforation.^24^, ^25^ Recently, several studies have reported that LECS is effective for duodenal tumors^10^, ^11^ and could be an ideal alternative to ESD in terms of safety and feasibility.^26^ However, no study has evaluated the efficacy of LECS limited to DNETs. In the present study, we mainly applied ER for DNETs <5 mm and LECS for DNETs 5–10 mm. While the R0 resection rate of ER for DNETs has been reported as approximately 40–60%,^13^, ^14^ it was found to be more promising in our study (89%). This could be attributed to the therapeutic strategy in which the ER was adapted for in this study, namely DNETs <5 mm. The complication rate of ER for DNETs was 11%, which was comparable with those reported in previous studies (5–9%).^13^, ^14^ LECS achieved R0 resection in all cases, and the complication rate was very low. ER was considered appropriate for the treatment of DNETs <5 mm. Although the procedural time, time to first oral intake, and length of hospital stay were longer than ER, LECS was suggested to be a safe and feasible treatment option for DNETs 5–10 mm.

This study has several limitations. First, this was a single‐center retrospective study that investigated the outcomes of local resection for DNETs and the risk factors for LNM. There were potential biases when selecting the treatment methods and retrospectively assessing the outcomes. Second, the required follow‐up period for assessing LNM was at least 12 months, and LNM in the locally resected cases without lymph node dissection was assessed using only CT. This could lead to an underestimation of LNM because CT is not the most appropriate modality for assessing LNM, and 12 months is too short to appropriately confirm the absence of LNM. Third, the number of LNM‐positive cases was small, which made it difficult to conduct a multivariate analysis. Therefore, a multicenter study involving a larger number of patients with a sufficient follow‐up period is needed. Despite these limitations, this study is valuable for the evaluation of treatment methods for DNETs based on the risk factors for LNM and the outcomes of local resection.

In conclusion, we found the significant risk factors for LNM of DNETs to be tumor size >10 mm, positive LVI, and 0‐Is morphology. Our study demonstrated that ER could serve as a safe and feasible treatment option for DNETs <5 mm and LECS for DNETs 5–10 mm.

## References

[1] Ito T , Sasano H , Tanaka M *et al*. Epidemiological study of gastroenteropancreatic neuroendocrine tumors in Japan. J. Gastroenterol. 2010; 45: 234–43. 20058030 10.1007/s00535-009-0194-8

[2] Modlin IM , Lye KD , Kidd M . A 5‐decade analysis of 13,715 carcinoid tumors. Cancer. 2003; 97: 934–59. 12569593 10.1002/cncr.11105

[3] Ahmed M . Gastrointestinal neuroendocrine tumors in 2020. World J. Gastrointest. Oncol. 2020; 12: 791–807. 32879660 10.4251/wjgo.v12.i8.791 PMC7443843

[4] Scherübl H , Jensen RT , Cadiot G , Stölzel U , Klöppel G . Neuroendocrine tumors of the small bowels are on the rise: early aspects and management. World J. Gastorointest. Endosc. 2010; 2: 325–34. 10.4253/wjge.v2.i10.325 PMC2998818 21160582

[5] Delle Fave G , O'Toole D , Sundin A *et al*. ENETS consensus guidelines update for gastroduodenal neuroendocrine neoplasms. Neuroendocrinology. 2016; 103: 119–24. 26784901 10.1159/000443168

[6] NCCN clinical Practice guidelines in oncology (NCCN guidelines). Neuroendocrine tumors. Version 2. 2016. Available from URL: http://www.nccn.org/professionals/physician_gls/pdf/neuroendocrine.pdf

[7] Clinical practice guidelines for gastroenteropancreatic neuroendocrine neoplasms 2019. (In Japanese.) Available from URL: http://www.nccn.org/professionals/physician_gls/pdf/neuroendocrine.pdf

[8] Hara Y , Goda K , Dobashi A *et al*. Short‐ and long‐term outcomes of endoscopically treated superficial non‐ampullary duodenal epithelial tumors. World J. Gastroenterol. 2019; 25: 707–18. 30783374 10.3748/wjg.v25.i6.707 PMC6378536

[9] Shibagaki K , Ishimura N , Kinoshita Y . Endoscopic submucosal dissection for duodenal tumors. Ann. Transl. Med. 2017; 5: 188. 28616403 10.21037/atm.2017.03.63 PMC5464942

[10] Ichikawa D , Komatsu S , Dohi O *et al*. Laparoscopic and endoscopic co‐operative surgery for non‐ampullary duodenal tumors. World J. Gastroenterol. 2016; 22: 10424–31. 28058023 10.3748/wjg.v22.i47.10424 PMC5175255

[11] Nunobe S , Ri M , Yamazaki K *et al*. Safety and feasibility of laparoscopic and endoscopic cooperative surgery for duodenal neoplasm: a retrospective multicenter study. Endoscopy. Published online 2 Dec 2020. 10.1055/a-1327-5939. 33264810

[12] Nishio M , Hirasawa K , Ozeki Y *et al*. Short‐ and long‐term outcomes of endoscopic submucosal dissection for non‐ampullary duodenal neuroendocrine tumors. Ann. Gastroenterol. 2020; 33: 265–71. 32382229 10.20524/aog.2020.0477 PMC7196614

[13] Harnden I , Walker R , Balmadrid B *et al*. Endoscopic mucosal resection of duodenal carcinoid tumors: a single tertiary care center experience. Gastroenterol. Hepatol. Open Access. 2015; 3: 00075.

[14] Kim GH , Kim JI , Jeon SW *et al*. Endoscopic resection for duodenal carcinoid tumors: a multicenter, retrospective study. J. Gastroenterol. Hepatol. 2014; 29: 318–24. 24117946 10.1111/jgh.12390

[15] No Authors . The Paris endoscopic classification of superficial neoplastic lesions: esophagus, stomach, and colon: November 30 to December 1, 2002. Gastrointest. Endosc. 2003; 58: S3–43. 14652541 10.1016/s0016-5107(03)02159-x

[16] Nagtegaal ID , Odze RD, Klimstra D *et al*. The 2019 WHO classification of tumours of the digestive system. Histopathology. 2020; 76: 182–8. 10.1111/his.13975 PMC7003895 31433515

[17] Dindo D , Demartines N , Clavien PA . Classification of surgical complications: a new proposal with evaluation in a cohort of 6336 patients and results of a survey. Ann. Surg. 2004; 240: 205–13. 15273542 10.1097/01.sla.0000133083.54934.ae PMC1360123

[18] Kanda Y . Investigation of the freely available easy‐to‐use software ‘EZR’ for medical statistics. Bone Marrow Transplant. 2013; 48: 452–8. 23208313 10.1038/bmt.2012.244 PMC3590441

[19] Sato Y , Hashimoto S , Mizuno K , Takeuchi M , Terai S . Management of gastric and duodenal neuroendocrine tumors. World J. Gastroenterol. 2016; 22: 6817–28. 27570419 10.3748/wjg.v22.i30.6817 PMC4974581

[20] Park SG , Lee BE , Kim GH *et al*. Risk factors for lymph node metastasis in duodenal neuroendocrine tumors. Medicine (Baltimore). 2019; 98: e15885. 31169696 10.1097/MD.0000000000015885 PMC6571284

[21] Vanoli A , La Rosa S , Klersy C *et al*. Four neuroendocrine tumor types and neuroendocrine carcinoma of the duodenum: analysis of 203 cases. Neuroendocrinology. 2017; 104: 112–25. 26910321 10.1159/000444803

[22] Carter JT , Grenert JP , Rubenstein L , Stewart L , Way LW . Neuroendocrine tumors of the ampulla of Vater: biological behavior and surgical management. Arch. Surg. 2009; 144: 527–31. 19528385 10.1001/archsurg.2009.80

[23] Hatta W , Koike T , Iijima K *et al*. The risk factors for metastasis in non‐ampullary duodenal neuroendocrine tumors measuring 20 mm or less in diameter. Digestion. 2017; 95: 201–9. 28315861 10.1159/000459619

[24] Hoteya S , Kaise M , Iizuka T *et al*. Delayed bleeding after endoscopic submucosal dissection for non‐ampullary superficial duodenal neoplasias might be prevented by prophylactic endoscopic closure: analysis of risk factors. Dig. Endosc. 2015; 27: 323–30. 25186455 10.1111/den.12377

[25] Matsumoto S , Miyatani H , Yoshida Y , Nokubi M . Duodenal carcinoid tumors: 5 cases treated by endoscopic submucosal dissection. Gastrointest. Endosc. 2011; 74: 1152–6. 21944312 10.1016/j.gie.2011.07.029

[26] Ojima T , Nakamori M , Nakamura M *et al*. Laparoscopic and endoscopic cooperative surgery versus endoscopic submucosal dissection for the treatment of low‐risk tumors of the duodenum. J. Gastrointest. Surg. 2018; 22: 935–40. 29352442 10.1007/s11605-018-3680-6

